# Differential Absorbance and PPG-Based Non-Invasive Blood Glucose Measurement Using Spatiotemporal Multimodal Fused LSTM Model

**DOI:** 10.3390/s25175260

**Published:** 2025-08-24

**Authors:** Jinxiu Cheng, Pengfei Xie, Huimeng Zhao, Zhong Ji

**Affiliations:** Key Laboratory of Biorheological Science and Technology of Ministry of Education, College of Bioengineering, Chongqing University, Chongqing 400044, China; chengjinxiu@stu.cqu.edu.cn (J.C.); 202219131117@stu.cqu.edu.cn (P.X.); zhaohuimeng@stu.cqu.edu.cn (H.Z.)

**Keywords:** non-invasive blood glucose measurement, differential absorbance, PPG, LSTM, multimodal fusion, spatiotemporal modeling

## Abstract

Blood glucose monitoring is crucial for the daily management of diabetic patients. In this study, we developed a differential absorbance and photoplethysmography (PPG)-based non-invasive blood glucose measurement system (NIBGMS) using visible–near-infrared (Vis-NIR) light. Three light-emitting diodes (LEDs) (625 nm, 850 nm, and 940 nm) and three photodetectors (PDs) with different source–detector separation distances were used to detect the differential absorbance of tissues at different depths and PPG signals of the index finger. A spatiotemporal multimodal fused long short-term memory (STMF-LSTM) model was developed to improve the prediction accuracy of blood glucose levels by multimodal fusion of optical spatial information (differential absorbance and PPG signals) and glucose temporal information. The validity of the NIBGMS was preliminarily verified using multilayer perceptron (MLP), support vector regression (SVR), random forest regression (RFR), and extreme gradient boosting (XG Boost) models on datasets collected from 15 non-diabetic subjects and 3 type-2 diabetic subjects, with a total of 805 samples. Additionally, a continuous dataset consisting 272 samples from four non-diabetic subjects was used to validate the developed STMF-LSTM model. The results demonstrate that the STMF-LSTM model indicated improved prediction performance with a root mean square error (RMSE) of 0.811 mmol/L and a percentage of 100% for Parkes error grid analysis (EGA) Zone A and B in 8-fold cross validation. Therefore, the developed NIBGMS and STMF-LSTM model show potential in practical non-invasive blood glucose monitoring.

## 1. Introduction

Diabetes is a chronic and irreversible metabolic disease characterized by abnormal blood glucose concentrations. Sustained hyperglycemia can cause various complications including diabetic retinopathy, heart disease, stroke, kidney damage, blindness, and neuropathy [1]. Individuals diagnosed with diabetes require measurement of their blood glucose levels in a regular way and control of them within the normal range to avoid suffering from complications. Currently, self-monitoring of blood glucose is mainly accomplished by blood glucose meters and continuous glucose monitors [2]. However, blood glucose detections are still not carried out as often as recommended due to the inconvenience, invasiveness, and high cost of current commercially available methods [3]. Therefore, there is a critical need for a readily achievable method that measures blood glucose levels with minimal discomfort, high accuracy, and low cost.

Photoplethysmography (PPG) is an optical-based, non-invasive technique for detecting variations in blood volume within the tissue’s microvascular bed [4]. The sensor consists a pair of light transmitter and detector. Light is emitted into the skin, and the intensity of transmitted or backscattered light detected by the light detector varies with blood volume due to the absorbent properties of blood to visible and infrared light. PPG has long been widely used for the measurement of heart rate (HR), heart rate variability (HRV), peripheral blood oxygen saturation [4], blood pressure [5,6], and blood glucose [7,8,9,10], as well as the detection of hypertension [11], diabetes [12], arterial stiffness [13], and coronary heart disease [14]. Previous studies indicated that hyperglycemia directly leads to elevated blood viscosity, which affects the shape of the PPG waveform [5,15]. Furthermore, the literature revealed that HRV has a negative correlation with blood glucose levels [16]. Effective information related to blood glucose concentrations can be extracted by analyzing the morphological and temporal features of PPG signals. Advancements in technology and the widespread adoption of smart devices, such as smartphones, smart watches, and fitness trackers, have facilitated the measurement of PPG, providing an opportunity for developing non-invasive monitoring method of blood glucose based on smart devices.

There have been numerous studies on PPG signal-based non-invasive blood glucose measurement methods. Monte-Moreno [5] extracted features such as autoregressive (AR) coefficients, Kaiser–Teager energy, heart rate, oxygen saturation range, spectral entropy statistics, and individual information to estimate blood glucose levels. The best performance was obtained from the random forest, with 87.7% of the points falling into region A of the Clarke error grid analysis (EGA). Hina et al. [7] detected PPG signals using a single wavelength near-infrared light (940 nm) and extracted the features including logarithmic energy entropy, Kaiser-Teager energy, spectral entropy, and AR coefficients of the PPG signals. A support vector machine (SVM) was developed to predict blood glucose levels; the results demonstrated that 95% of the prediction results lie within region A of the Clarke EGA, with a coefficient of determination (R^2^) of 0.937. Tsai et al. [17] collected PPG signals from the fingers and wrists using a wearable health device, Glutrac. A total of 30 features were extracted from the denoised signal and its first derivative. An overall accuracy of 80% was achieved using multiple decision trees, with significant variations in individual accuracy. Du et al. [18] developed a non-invasive fingertip blood glucose detection system using PPG, multiple near-infrared (NIR) wavelengths, and photodetectors (PDs). The root mean square error (RMSE) of 0.242 mmol/L and 100% of the prediction results in area A of the Parkes error grid were achieved from 10 non-diabetic subjects using a deep learning (DL) model based on a DenseNet integrating residual link and multiple self-attention mechanisms.

As we all know, blood glucose concentrations fluctuate with a certain regularity during a day. Data-driven models can predict blood glucose levels based on historical values without requiring prior knowledge. Recurrent neural networks (RNNs) are considered more effective models in terms of dealing with sequential data than traditional machine learning methods, which use previous outputs as inputs of the current step. Long short-term memory (LSTM), a variant of RNN, solves the gradient explosion and vanishing problems of RNN through a memory cell, which is updated at each step to store and output information [19]. Aliberti et al. [20] developed and compared nonlinear autoregressive (NAR) neural networks with LSTM networks. The results indicated that the NAR performed well only for 30 min predictions, whereas the LSTM obtained desirable performance both for 30 min, 60 min, and longer prediction horizons. Kim et al. [21] tested a simple RNN, gated recurrent unit (GRU), and LSTM on 20 in-hospital patients. The average RMSE and mean absolute percentage error (MAPE) of GRU were 21.5 mg/dL (1.19 mmol/L) and 11.1%, respectively. Deng et al. [22] achieved RMSE of 19.08 mg/dL (1.06 mmol/L) and 33.8 mg/dL (1.88 mmol/L) for 30 min and 60 min prediction horizons using a convolutional neural network (CNN) and transfer learning.

So far, there have been a large number of studies achieving non-invasive glucose prediction based on PPG signals or data-driven approaches with favorable performance. Nevertheless, several limitations still exist. First, the specificity of PPG signals for glucose detection is limited, and only using PPG signals is insufficient to ensure accuracy and generalizability. It is necessary to introduce direct physiological constraints related to blood glucose. Second, many studies based on data-driven methods only used historical glucose values as learning data without considering the physiological constraints. The prediction accuracy of data-driven methods generally decreases as the prediction horizon increases. Accurate prediction of blood glucose values can hardly be accomplished by merely data-driven methods. Thus, in this study, a wearable non-invasive blood glucose measurement system (NIBGMS) with a low cost was developed to simultaneously collect PPG signals and differential absorbance. Additionally, a multimodal fusion strategy was adopted to fuse optical spatial information (differential absorbance and PPG signals) and blood glucose temporal information. A spatiotemporal multimodal fused long short-term memory (STMF-LSTM) model was developed to realize more accurate and generalized prediction of blood glucose concentrations. The results demonstrate that an accurate non-invasive blood glucose measurement method has been achieved using the developed NIBGMS and STMF-LSTM model.

## 2. Materials and Methods

### 2.1. Design of the Non-Invasive Blood Glucose Measurement System

Variation in blood glucose levels affects the light intensity absorbed by blood vessels in body tissue. Optical detection is an effective but challenging method for non-invasive blood glucose measurement. In this study, the fingertip was selected as the measurement site owing to its high capillary density and convenience of implementation. Wavelengths greater than 1000 nm exhibit strong absorption with water. Detection wavelengths were selected within the range of 610–1000 nm, avoiding the strong absorption range of water and ensuring sufficient tissue penetration depth. The wavelength of 939 nm is one of the absorption peaks of glucose in the higher overtone region [23]. In addition, wavelengths at 625 nm and 850 nm have lower water absorption and avoid the strong absorption range of hemoglobin. Therefore, three light-emitting diodes (LEDs) with different wavelengths (625 nm, 850 nm, and 940 nm) in the visible–near-infrared (Vis-NIR) region were used to obtain wavelength resolution of absorbance information.

**Figure 1:**
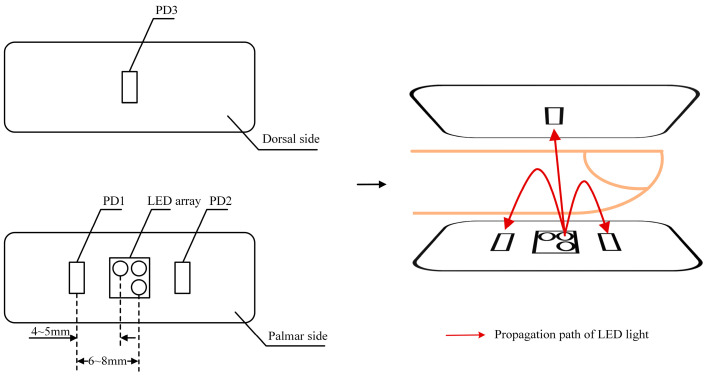
Distribution diagram of LEDs and PDs in finger clip sensor.

For non-invasive blood glucose measurement, the key is to obtain absorbance information of the subcutaneous tissue layer, which is rich in capillaries and therefore highly correlated with blood glucose concentrations. Studies on light–tissue interactions showed that the penetration depth through the tissue gradually increases with the source–detector (LED-PD) separation distance [24,25]. The absorbance information of deeper tissues can be detected by PD with larger source–detector separation distance. Utilizing PDs with different source–detector separations enables the detection of absorbance information arising from various depths of the tissues, thereby providing depth resolution of absorbance information. Therefore, a finger clip sensor with LEDs and PDs distributed as in Figure 1 was designed.

An LED array composed of three LEDs (625 nm, 850 nm, and 940 nm) is located on the palmar side of the finger, and the LEDs are illuminated in turn. Two PDs (PD1, PD2) with source–detector separation distances of 4–5 mm and 6–8 mm, respectively, are used to detect reflected light with different penetration depths. The third PD (PD3) is distributed on the dorsal side of the finger to detect transmitted light. Differential absorbances were calculated from detected light intensities of different optical pathways to extract absorbance information of the subcutaneous tissue layer. Taking one of the light sources of the wavelength as an example, the differential absorbances were calculated as follows:

(1) $$\begin{array}{l} \Delta A_{near-far}(\lambda )\\ =A_{far}(\lambda )-A_{near}(\lambda )\\ =\log\frac{I_{0}(\lambda )}{I_{far}(\lambda )}-\log\frac{I_{0}(\lambda )}{I_{near}(\lambda )}\\ =\log\frac{I_{near}(\lambda )}{I_{far}(\lambda )}. \end{array}$$

(2) $$\begin{array}{l} \Delta A_{near-trans}(\lambda )\\ =A_{trans}(\lambda )-A_{near}(\lambda )\\ =\log\frac{I_{0}(\lambda )}{I_{trans}(\lambda )}-\log\frac{I_{0}(\lambda )}{I_{near}(\lambda )}\\ =\log\frac{I_{near}(\lambda )}{I_{trans}(\lambda )}. \end{array}$$

where $I_{near}(\lambda )$ and $I_{far}(\lambda )$ are the detected light intensities at the near and far ends relative to the currently selected LED, respectively. $I_{trans}(\lambda )$ is the detected light intensity at the transmitted end. $I_{0}(\lambda )$ is the incident light intensity of the respective wavelength (λ). The differential absorbances corresponding to each of the three LEDs were calculated as described above.

**Figure 2:**
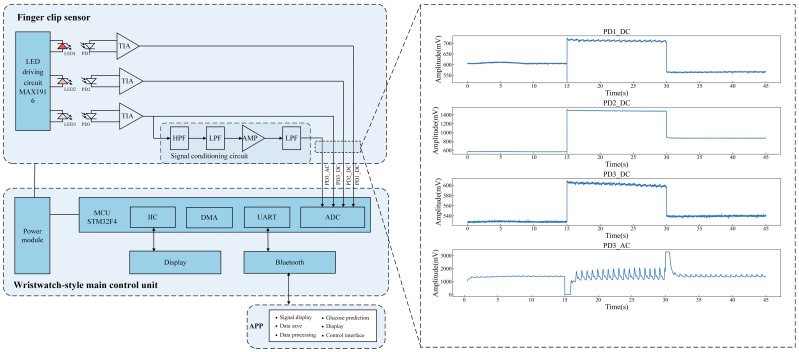
Schematic diagram of the NIBGMS.

**Figure 3:**
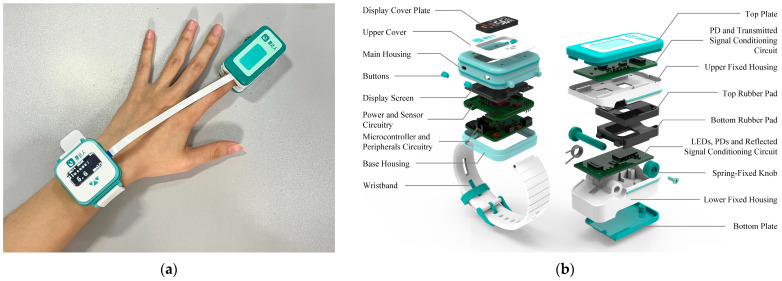
NIBGMS developed in this study. (**a**) The actual prototype diagram of NIBGM; (**b**) the exploded view of NIBGMS. The system consists of a wristwatch-style main control unit (**left**) and a finger clip sensor (**right**).

The block diagram of the developed wearable non-invasive blood glucose measurement system (NIBGMS) is shown in Figure 2. The system consists of a wristwatch-style main control unit and a finger clip sensor. The actual prototype diagram and exploded view of the system are depicted in Figure 3a,b, respectively.

In the finger clip sensor, three LEDs are controlled by three LED drivers and alternately activated. The driving current is 20 mA. The PDs can respond in the spectral range of sensitivity of 350 nm to 1070 nm. The optical sensor module used in our study is custom-designed using commercial off-the-shelf components. We developed this sensor in-house to meet the specific requirements of our research. The specifications of the LEDs and PDs are listed in Table 1. The sensor is encapsulated in a finger clip casing, with overall size of 55 mm × 30 mm × 30 mm. When each LED is sequentially activated, three channels of current signals are detected from three PDs, corresponding to light intensity after absorption of three paths (near reflection, far reflection, and transmission). The transimpedance amplifier (TIA) circuits convert the current signals output from PDs into corresponding voltage signals. In addition, the voltage signal from PD-3 is synchronously fed into an analog signal processing circuit to extract the transmitted PPG signal. As shown in Figure 2, the analog signal processing circuit consists of a first-order passive high-pass filter (HPF) with a cutoff frequency of 0.15 Hz to remove the DC component of the signal, a second-order active low-pass filter (LPF) with a cutoff frequency of 15 Hz to eliminate high-frequency noise, a voltage boost and amplification circuit to adjust the signal amplitude to the range of the microcontroller analog-to-digital converter (ADC), and a first-order passive low-pass filter (LPF) with a cutoff frequency of 25 Hz for smoothing. Signals acquired from each channel are shown in Figure 2. The X-axis represents time, and the signals are detected when the 625 nm, 850 nm, and 940 nm LEDs are sequentially activated for 15 s. There are some outliers around 15 s and 30 s, which were affected by light source switching. The stable portions of the signals, situated between these intervals, were utilized for subsequent signal analysis. PD1_DC, PD2_DC, and PD3_DC are the light intensity signals from different light propagation paths detected by the three PDs at the near, far, and transmission ends, respectively. When an LED is illuminated, the signal amplitudes detected from three PDs exhibit significant differences, corresponding to the light intensity after absorption from different optical propagation paths. PD3_AC is the transmitted PPG signal detected by PD at the transmission end. Repeated experiments were performed on 3 subjects, with each subject conducting 10 consecutive measurements under the same conditions. The maximum type A uncertainty of the PD sensors was 0.026 (Appendix A).

Another major component of the system is the main control unit. This unit includes a connector for flexible printed circuit wires, a power switch, and buttons for resetting the device and starting/stopping measurements. The STM32F4 microcontroller (STMicroelectronics NV, Geneva, Switzerland) is used as the processing unit for controlling the entire system. This microcontroller integrates a 12-bit ADC for signal conversion. Analog signals are converted into digital signals using the ADC. The sampling rate is 200 Hz. The control unit is wrapped in a wristwatch-type casing with a compact size (45 mm × 45 mm × 20 mm). The system is powered by a rechargeable battery pack (300 mAh, 3.7 V lithium polymer) to facilitate portable measurements.

A mobile application was developed using Android Studio. The application has buttons to connect the developed system via Bluetooth, initiate signal acquisition, and enable data storage. The collected data are transmitted via Bluetooth to the developed mobile application. After signal acquisition, the data are stored as a comma-separated value (CSV) file on the mobile device, and the predicted blood glucose value of the developed model is displayed to the application user.

### 2.2. Experiments

In this study, 15 non-diabetic subjects and 3 type-2 diabetic subjects were enrolled. The eligibility criteria for subjects included adults aged 18 to 80 years, regardless of gender. For the enrolment of type-2 diabetic subjects, it was required that no acute complications of diabetes within 3 months before enrolment, or severe chronic complications of diabetes and comorbidities, were observed. The demographics of the subjects are listed in Table 2. Informed consent was obtained from all subjects enrolled in this study prior to data collection. This study was approved by the Ethics Committee of Chongqing University Cancer Hospital (Ethics Approval No. 2020055). Abbot’s Freestyle Libre (Abbott Diabetes Care Ltd., Dublin, Ireland) continuous glucose monitors (CGMs) and OneTouch Verio Flex (LifeScan Europe GmbH, Inverness, UK) blood glucose meters were used to obtain reference blood glucose levels. Specifications of these two blood glucose reference instrumentations are listed in Table A2 of Appendix B. A protocol was established for acquiring PPG and absorbance signals using the developed non-invasive blood glucose measurement system. Subjects were asked to fast for at least 8 h before fasting blood glucose levels were measured and to avoid strenuous physical activity on the day of the test. The detection site of the finger clip sensor was fixed as the index finger of the left hand to minimize the impact of structural differences. Subjects were instructed to sit still and rest their elbow on the desk during the experiment. In addition, before the measurement, subjects were instructed to soak their hands in warm water at 35–40 °C for a few minutes to ensure adequate peripheral blood flow. Data collection was primarily conducted before meals as well as during the two hours after meals. The specific number of samples collected each day varied among subjects due to individual differences. A total of 805 sets of signals with corresponding reference glucose values, which ranged from 3.9 to 29.1 mmol/L, were collected. This included 16 days of continuous data collected from 4 non-diabetic subjects.

### 2.3. LSTM Network

**Figure 4:**
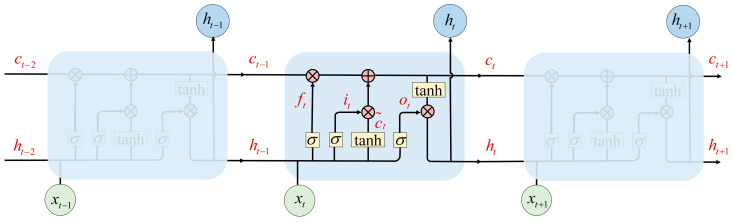
Internal structure of LSTM.

The long short-term memory (LSTM) network has been widely used in time series prediction, and overcomes the limitations of RNN in capturing long-term dependencies [19]. The structure of the LSTM is depicted in Figure 4. The cell state is the key component of LSTM, which flows through the entire chain. LSTM has the ability to add information to or remove information from the cell state, finely regulated by three gates:

(3) $$f_{t}=\sigma (W_{f}\cdot [h_{t-1},x_{t}]+b_{f})$$

(4) $$i_{t}=\sigma (W_{i}\cdot [h_{t-1},x_{t}]+b_{i})$$

(5) $$o_{t}=\sigma (W_{o}\cdot [h_{t-1},x_{t}]+b_{o})$$

where $f_{t},\;i_{t},\;o_{t}$ are the forget gate, input gate, and output gate, respectively, each of which is a nonlinear function activated by a weighted sum of the current input $x_{t}$ and previous output $h_{t-1}$; $W_{f},\;W_{i},\;W_{o}$ are the weight matrices of the forget gate, input gate, and output gate, respectively; $b_{f},\;b_{i},\;b_{o}$ are the biases of the forget gate, input gate and output gate, respectively.

The candidate cell state ${\tilde{c}}_{t}$ is calculated by the tanh function, which could be added to the new cell state; the expression is as follows:

(6) $${\tilde{c}}_{t}=\tanh\left(W_{c}\cdot [h_{t-1},x_{t}]+b_{c}\right)$$

where $W_{c}$ is the weight matrix and $b_{c}$ is the bias for the candidate cell state. Then, the old cell state $c_{t-1}$ is updated to the new cell state $c_{t}$ as follows:

(7) $$c_{t}=f_{t}*c_{t-1}+i_{t}*{\tilde{c}}_{t}$$

The forget gate $f_{t}$ decides how much information of the previous cell state $c_{t-1}$ to be thrown out. The input gate $i_{t}$ determines how much information of the candidate cell state ${\tilde{c}}_{t}$ is to be stored in the new cell state $c_{t}$. The final output of the LSTM $h_{t}$ is determined by the new cell state $c_{t}$ and output gate $o_{t}$, where the output gate decides what parts of the new cell state to be output; the specific expression is as follows:

(8) $$h_{t}=o_{t}*\tanh\left(c_{t}\right)$$

After the data passes through these three gates, the valuable information is output and the invalid information is discarded.

### 2.4. PPG Signal Processing and Feature Extraction

The wavelength of 940 nm has good penetration properties and a favorable correlation with blood glucose [26]. Therefore, transmitted PPG signals of 940 nm were used for feature extraction. Preprocessing of the PPG signal segments is essential for further feature extraction. A six-order low-pass Butterworth filter with a cutoff frequency of 15 Hz was applied to eliminate high-frequency noise from the recorded PPG signals. A cubic spline interpolation algorithm was used to remove baseline drifts of PPG signals. Then, the PPG signals were normalized to eliminate the influence of different signal amplitudes. The PPG signals after preprocessing were used to extract following features:

#### 2.4.1. Heart Rate

Instantaneous heart rate (HR) series was derived from the intervals between consecutive systolic peaks of the PPG signal. The mean value and standard deviation of the series were calculated as features $HR^{\mu }$ and $HR^{\sigma }$, respectively, where $HR^{\sigma }$ was used to reflected the heart rate variability.

#### 2.4.2. KTE and logE

**Figure 5:**
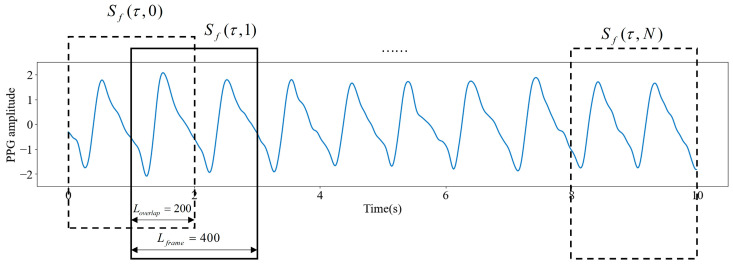
Frames of PPG signal segments.

Kaiser–Teager energy (KTE) and logarithmic energy entropy (logE) characterize the shape variations and energy profiles of PPG signals, reflecting the impact of blood glucose concentration variations on PPG waves indirectly. Mapping of these features with measured glucose levels using machine learning algorithms enables blood glucose prediction based on PPG signals. KTE is commonly calculated to reflect the energy profiles of periodic signals [27]. As shown in Figure 5, the PPG signal is divided into a frame array $S_{f}\left(\tau ,n\right)$; the number of samples in each frame is $L_{frame}=400$, which is twice the sampling rate; the overlap length between two adjacent frames is $L_{overlap}=200$; the number of frames of each PPG signal is N=9; the index n=1,2,…,N indicates the frame number of the frame array; and $\tau =1,2,\ldots ,L_{frame}$ denotes the sample number in each frame. By dividing the PPG signal into short frames, local features can be extracted while reducing the influence of signal amplitude variations due to breathing. In addition, the 50% overlap length between frames ensures signal continuity and avoids missing information at the frame boundaries. Hence, the KTE sequence can be computed at the frame level as follows:

(9) $$KTE(\tau ,n)=S_{f}^{2}(\tau ,n)-S_{f}(\tau +1,n)\times S_{f}(\tau -1,n)$$

Statistical measures of the KTE sequence were calculated as features, including mean $KTE^{\mu }$ and variance $KTE^{\sigma }$.

In addition, logE is a time-domain entropy measure computed from full-band energy spectra [28]. LogE is also computed at the frame level as given in (10):

(10) $$LogE(n)=Log(\sum_{\tau =1}^{L_{frame}}S_{f}^{2}(\tau ,n))$$

Statistical measures of the logE sequence were calculated as features, including mean $\log E^{\mu }$ and variance $\log E^{\sigma }$.

Finally, as shown in (11), an overall feature vector $x_{F}$ with a length of 12 was constructed by the above features and used as part of the inputs for the subsequent glucose prediction model.

(11) $$\begin{array}{l} x_{F}=[HR^{\mu },HR^{\sigma },KTE^{\mu },KTE^{\sigma },\log E^{\mu },\log E^{\sigma },\\ \Delta A_{near-far}(625\;\mathrm{nm}),\;\Delta A_{near-trans}(625\;\mathrm{nm}),\\ \Delta A_{near-far}(850\;\mathrm{nm}),\;\Delta A_{near-trans}(850\;\mathrm{nm}),\\ \Delta A_{near-far}(940\;\mathrm{nm}),\;\Delta A_{near-trans}(940\;\mathrm{nm})] \end{array}$$

### 2.5. Development of STMF-LSTM Model

**Figure 6:**
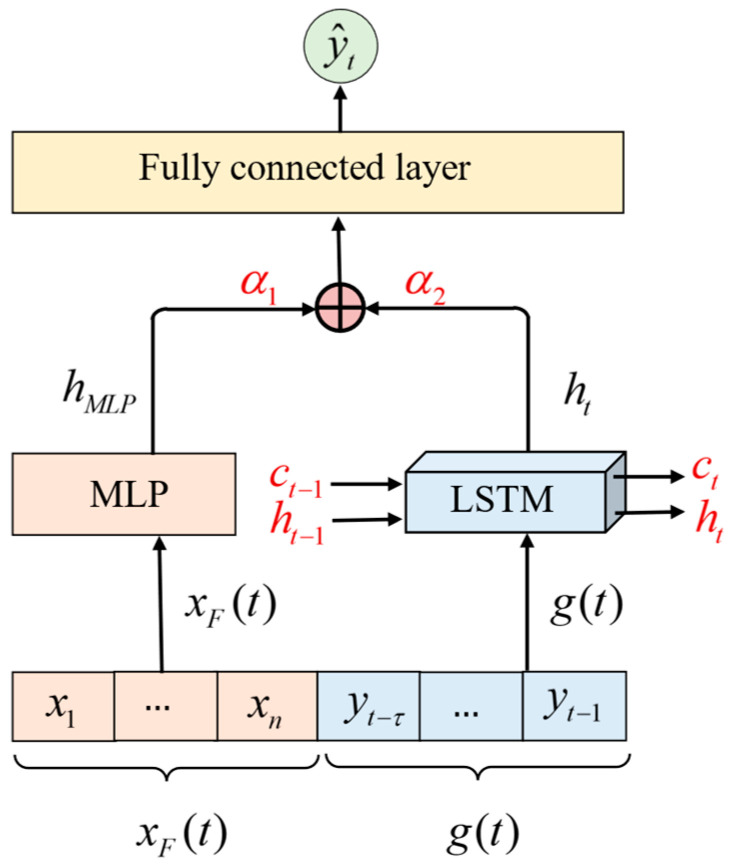
Schematic of the STMF-LSTM model.

A spatiotemporal multimodal fused long short-term memory (STMF-LSTM) model was developed to integrate optical spatial information and blood glucose temporal information. As shown in Figure 6, the multilayer perceptron (MLP) was integrated to process optical spatial information. The MLP maps the feature vector $x_{F}(t)$, consisting of differential absorbances and features extracted from PPG signals to a high-dimension representation $h_{MLP}$ that captures the nonlinear relationship. LSTM was employed to learn the temporal fluctuation information of glucose values g(t). The hidden state and cell state h(t), c(t) of LSTM are updated at each timestep. The learned temporal information is output through h(t), enabling the prediction of the current blood glucose value based on historical values g(t). A weighted combination fusion strategy was adopted to fuse optical spatial information and glucose temporal information. The hidden states of MLP and LSTM were fused in the fully connected layer with weights of $\alpha_{1}$ and $\alpha_{2}$, respectively. The weighting parameters $\alpha_{1}$ and $\alpha_{2}$ balance the contributions of the spatial and temporal information. Finally, the prediction value of blood glucose was achieved from the fully connected layer. The STMF-LSTM model integrates the strengths of LSTM in time series prediction and the advantages of MLP in fitting the relationship between input features and glucose values. This is particularly beneficial to leverage complementary information from optical features and historical glucose values, leading to improved accuracy in blood glucose prediction.

## 3. Results and Discussion

**Figure 7:**
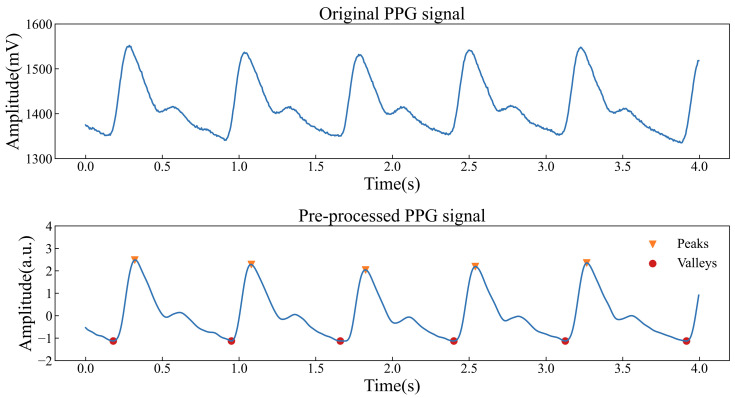
Original and preprocessed PPG signals.

Signal preprocessing was performed to remove invalid information from the PPG signals before feature extraction. As shown in Figure 7, the signal after preprocessing has obvious waveform characteristics and no redundant noise interference, so that feature points can be identified and feature parameters can be extracted accurately. After that, six PPG features were extracted from the preprocessed PPG signals and six absorbance differences were calculated. These 12 parameters formed the input feature vector $x_{F}(t)$. The input features and glucose levels were normalized using the Z-score normalization (ZSN) method [29], which scales the data using mean and standard deviation measures, ensuring that the resultant glucose levels and input features have zero means and unit variances.

### 3.1. Preliminary Validation of the NIBGMS

The MLP, support vector regression (SVR), random forest regression (RFR), and extreme gradient boosting (XG Boost) algorithms were developed to preliminarily validate the effectiveness of the NIBGMS using the diabetic and non-diabetic datasets, respectively. Each dataset was divided into 10 equal folds, and the 10-fold cross-validation was performed, with each fold in turn serving as the validation set to assess the performance of the models. The six PPG features and six absorbance differences were combined as the input features of model. The structure and training parameters of the models are presented in Table 3.

The root mean square error (RMSE), mean absolute error (MAE), and correlation coefficient (CORR) between the predicted and reference glucose values were calculated to evaluate the prediction performance of the models. Additionally, Parkes error grid analysis (EGA) [30] was also performed to visualize the consistency between the predicted and reference glucose values.

**Figure 8:**
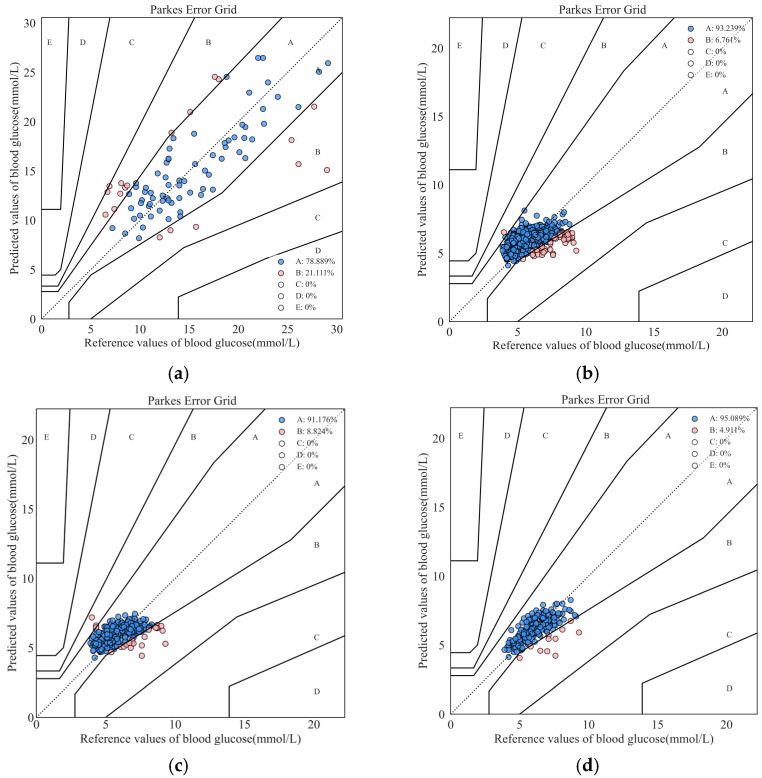
Parkes error grid analysis of (**a**) MLP on diabetic dataset; (**b**) MLP on non-diabetic dataset; (**c**) MLP on continuous dataset; (**d**) STMF-LSTM on continuous dataset.

The overall prediction performance of the models is shown in Table 4. The results showed that the MLP, SVR, RFR, and XG Boost models achieved favorable prediction results on both the diabetic dataset and non-diabetic dataset. All the predicted values of the models were distributed within regions A and B of the Parkes EGA. The prediction error of the diabetic dataset was higher than that of the non-diabetic dataset due to its larger variation range of blood glucose values. The prediction performance of the MLP model outperformed that of the SVR, RFR, and XG Boost models on both the diabetic dataset and non-diabetic dataset. The RMSE and CORR of the MLP model on the diabetic dataset were 3.784 mmol/L and 0.745, respectively, while those on the non-diabetic dataset were 0.953 mmol/L and 0.472, respectively. The results of the Parkes EGA of MLP models on diabetic and non-diabetic datasets are shown in Figure 8a,b. The percentage of predicted values falling into region A were 78.889% and 93.239%, respectively. The results demonstrated that the NIBGMS system developed in this study achieved favorable results on both diabetic and non-diabetic datasets, based on the simple MLP structure and few input features, validating the feasibility of the developed NIBGMS system for non-invasive glucose measurement.

### 3.2. Cross Validation of STMF-LSTM Model

The 8-fold cross validation was performed using the STMF-LSTM model on the continuous dataset, consisting of 272 samples of 16 days collected from four subjects. Each fold contained data of 2 days. The training dataset was created using the input features and glucose values. The glucose values were divided into input and output parts using the lag-time method. Considering that excessive invasive detection of blood glucose values should be avoided, the delay order τ was set to 1. The glucose value at the pervious time step, y(t−1), was used as the historical glucose value and combined with the input features to constitute the input vector, and y(t) was used as the reference glucose value at the present time step. The Adam optimization algorithm with a learning rate of 0.01 was used to train the STMF-LSTM models. The structure and training parameters of the STMF-LSTM model are presented in Table 3.

**Figure 9:**
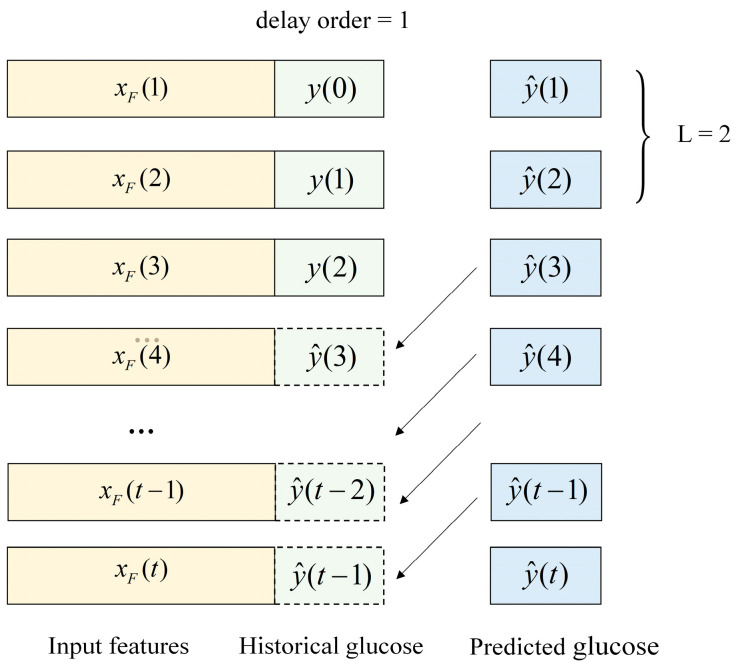
Overall description of the multi-step prediction.

Initially, the STMF-LSTM model was trained to predict the target glucose value for one step ahead, whereas at the validation stage, multi-step prediction was performed on the well-trained STMF-LSTM model using a recursive method. As described in Figure 9, the predicted value was reused as the historical glucose value to achieve prediction of the step afterwards. For example, after achieving the predicted value for the current time step t−1, this predicted value was reused as the historical glucose value and, together with feature vector $x_{F}(t)$ extracted from optical signals, constituted the input for the next time step t. This recursive approach was adopted to align with practical prediction processes, where the real blood glucose values are not available and need to be predicted step by step. Since each prediction is based on the predicted value at the previous time step, any error in the previous predictions can propagate and potentially amplify in subsequent steps. To compensate for this deficiency, optical spatial information was fused in the STMF-LSTM model through a spatiotemporal multimodal fusion strategy, thereby correcting the accumulated errors and improving the accuracy of the long-term predictions.

According to the results of the 8-fold cross validation, the overall prediction performance of the STMF-LSTM models on the continuous dataset is shown in Table 4. It can be concluded that the STMF-LSTM model had the lowest RMSE and MAE, which were 0.811 mmol/L and 0.620 mmol/L, respectively. Additionally, CORR of the STMF-LSTM model increased to 0.678, significantly surpassing that of the MLP, SVR, RFR, and XG Boost models. The result of the Parkes EGA of MLP and STMF-LSTM models on the continuous dataset are shown in Figure 8c,d, respectively. It can be observed that all the predicted values of the MLP and STMF-LSTM models were distributed within regions A and B. However, for the continuous dataset, the predicted values of the STMF-LSTM model were more concentrated in region A and showed a higher correlation with the reference values compared to the MLP model. The percentage of predicted results falling into region A increased to 95.089%. This demonstrates that the prediction accuracy of glucose values was significantly improved by the spatiotemporal multimodal fusion strategy, attributed to the specialty of LSTM in time series prediction and the strength of MLP model in fitting input features and glucose values.

**Figure 10:**
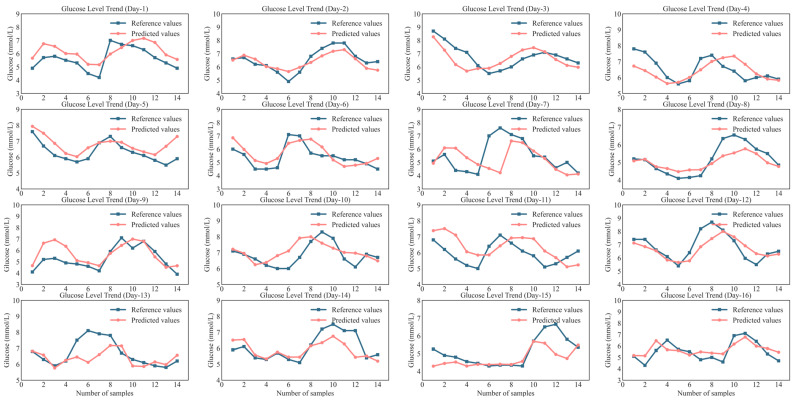
Prediction values and reference values of STMF-LSTM models.

The curves of the predicted and reference glucose values for 16 days are shown in Figure 10. The label on the X-axis is the sample number; for example, *i* represents the *i-th* predicted sample data of the day. It can be observed that the predicted values of the STMF-LSTM model were in good agreement with the reference values. However, some of the predictions of individual sequences had a certain degree of delay, since CGM measures glucose concentration in subcutaneous interstitial fluid rather than blood [31]. Glucose diffusion from capillaries to interstitial fluid takes 5–15 min, resulting in CGM values lagging behind fingertip blood glucose. Differences in the diffusion time between individuals may result in varying degrees of delay in the prediction results. Despite some delays in some predictions, the developed NIBGMS and STMF-LSTM model provide users with a reference for glucose fluctuation trends during blood glucose management. This enables further derivation of blood glucose variability, which is a significant and clinically meaningful glycemic metric that can facilitate clinicians in risk stratification and treatment decisions [32].

### 3.3. Ablation Experiment

Ablation experiments were performed to assess the effectiveness of the extracted differential absorbance and the developed STMF-LSTM model in this study. The methods and results of different ablation experiments are presented in Table 5. In method A, the MLP model was employed as the base model, with only features extracted from 940 nm-transmitted PPG signals as inputs. As for method B, in addition to the features extracted from PPG signals, the calculated differential absorbance values were also added as inputs to the MLP model. Method C introduced the STMF-LSTM model, integrating features extracted from 940 nm-transmitted PPG signals, the calculated differential absorbance values, and the glucose temporal information. Both methods were validated on the continuous dataset. The structure and training parameters of the models are presented in Table 3. Method A showed limited prediction performance compared to the other two methods. With the addition of differential absorbance, the prediction performance of method B improved accordingly, indicating that the differential absorbance complemented the model with more glucose concentration-related information. The developed STMF-LSTM model in method C outperformed the other two methods by incorporating blood glucose temporal fluctuation information. This highlights the effectiveness of multimodal fusion of optical spatial information and blood glucose temporal fluctuation information, thereby enhancing prediction performance of the model.

### 3.4. Comparison with Previous Studies

Table 6 presents a comparative analysis of the prediction performance of the proposed method in this study with that reported in several related studies over the past few years. Given that different studies utilized datasets with varying ranges of glucose values, mean absolute relative difference (MARD) was chosen as the primary parameter for a fair comparison. Kim et al. [21] employed a purely data-driven approach using historical glucose values for blood glucose prediction without incorporating glucose-specific physiological information, achieving an MARD of 11.1%. Ali et al. [33] achieved an MARD of 17.88% using PPG signals based on a cascaded bidirectional long short-term memory (BiLSTM) network. Chowdhury et al. [34] designed a neural network that integrated multimodal signals, including PPG, electrodermal activity (EDA), skin temperature (ST), and user-provided food logs, and obtained an MRAD of 12.57%. Du et al. [18] adopted PPG signals of five wavelengths (850 nm, 930 nm, 1050 nm, 1200 nm, and 1300 nm) and achieved optimal prediction performance thorough a DenseNet-ResNet hybrid architecture-based CBGnet, with a minimum MARD of 5.13%. The STMF-LSTM mode developed in our study integrated optical spatial information and glucose temporal information, and demonstrated suboptimal prediction performance with an MARD of 10.122%. However, our study only used three wavelengths, and the range of blood glucose values was also expanded to a certain extent. Moreover, the compact design of our system and comprehensive functions, such as Bluetooth connectivity with mobile applications and real-time display of signals and test results on a smartphone, greatly enhance the user experience and make it more user friendly. The developed system integrated an internal lithium-ion battery, eliminating the need for external power and thereby enhancing portability. This balance between performance and deployment positions our system favorably for home-based applications, particularly in resource-limited settings.

## 4. Conclusions

In this study, we developed a wearable non-invasive blood glucose measurement system (NIBGMS) using Vis-NIR light. The system detected differential absorbance information of three LEDs (625 nm, 850 nm, and 940 nm) through three distinct propagation paths, along with PPG signals of 940 nm. The differential absorbances were calculated and the features were extracted from PPG signals to predict glucose values. A spatiotemporal multimodal fused long short-term memory (STMF-LSTM) model was developed to enhance prediction accuracy, which fused multimodal information (optical spatial information and glucose temporal information) and integrated the strengths of LSTM in time series prediction and the advantages of MLP in fitting the relationship between input features and glucose values. The 8-fold cross validation results of the continuous dataset demonstrated that the STMF-LSTM mode had more accurate and robust prediction performance. Therefore, the proposed wearable NIBGMS and the developed STMF-LSTM model provide a feasible solution for accurate non-invasive blood glucose measurement with low cost.

However, in this study, to validate the feasibility of the developed system and the prediction model, the data acquisition was performed with subjects in the condition of rest, avoiding hand movements that potentially affect signal quality. The impact of motion artifacts was not investigated in this work. Therefore, in future work, various types of movements during ambulatory monitoring should be considered to develop a robust non-invasive blood glucose monitoring system. In addition, the contact pressure between the sensor and the detected finger also has an effect on the PPG waveform. Hence, a contact pressure detection module should be integrated to investigate the influence of contact pressure on the accuracy of non-invasive blood glucose measurement. Furthermore, factors such as food intake or calorie consumption, and physiology parameters (e.g., systolic and diastolic blood pressure), also significantly affect blood glucose levels. Last but not least, the current dataset has a relatively small sample size and is skewed towards non-diabetic individuals. The distribution of blood glucose values is imbalanced, concentrated in the middle range, with the number of samples with high and low blood glucose values typically fewer than those in the middle range. This may indeed limit the model’s generalization and robustness across different populations and clinical scenarios, especially in predicting extreme blood glucose values such as in hypoglycemia and hyperglycemia. In the future, additional samples should be collected from a larger and balanced cohort of subjects (including both hypoglycemic and hyperglycemic individuals). Additionally, data balance and augmenting methods such as the synthetic minority over-sampling technique (SMOTE) and generative adversarial networks (GANs) should be introduced, and more effective signal processing methods and complex algorithms such as deep neural networks are expected to be introduced. This will enable more effective utilization of the complementary information from different modalities, thereby improving the accuracy, robustness, and generalization of non-invasive glucose prediction.

## Figures and Tables

**Table 1:** Specifications of the LEDs and PDs.

Parameters	LEDs			PDs
	625 nm	850 nm	940 nm	
Type	CY-C1608SURC-T4	CY-C1608QTIR85-T1	CY-C1608QTIR94-T1	VEMD1060X01
Manufacturer	Shenzhen Chaoyue Photoelectric Co., Ltd., Shenzhen, China	Shenzhen Chaoyue Photoelectric Co., Ltd., Shenzhen, China	Shenzhen Chaoyue Photoelectric Co., Ltd., Shenzhen, China	Vishay Intertechnology, Inc., Malvern, PA, USA
Package form	0603	0603	0603	0805
Peak wave length (nm)	625	850	940	-
Forward voltage (V)	1.7–2.4	1.2	1.2	-
Spectral Range (nm)	-	-	-	350–1070 nm
Wavelength of peak sensitivity (nm)	-	-	-	820
Radiant sensitive area (mm^2^)	-	-	-	0.23 mm^2^
Angle of half sensitivity (deg)	-	-	-	±70 deg

**Table 2:** Demographics of the subjects.

Physical Condition	Glucose (mmol/L)	Age (Years)	Gender (Male/ Female)	Number of Subjects	Number of Samples	Continuous Dataset (Days)	Reference Glucose Measurement Device
Type-2 diabetic	6.5–29.1	64–73	1/2	3	93		Freestyle Libre
Non-diabetic	3.9–9.3	21–45	9/6	15	712	16	Freestyle Libre/ OneTouch Verio Flex
Total	3.9–29.1	21–73	10/8	18	805	16	

**Table 3:** The structure and training parameters of the models.

Model	Parameters	Common Setting
MLP	Number of layers: 3 Hidden size: 25	Loss: MSE Optimizer: Adam Epoch: 50 Learning rate: 0.01
STMF-LSTM	Number of layers: {3,3} Hidden size: {25,25}	
SVR	Kernel: ‘rbf’ C: 10 gamma: 0.01 epsilon: 0.5	
RFR	n_estimators:100 max_depth: 5	
XG Boost	n_estimators:100 learning_rate: 0.1 max_depth: 5 subsample: 0.8 colsample_bytree: 0.8 reg_alpha: 0.5 reg_lambda: 0.5 min_child_weight: 3	

**Table 4:** The overall prediction performance of the models.

Dataset	Model	Parkes EGA (%)			RMSE (mmol/L)	MAE (mmol/L)	CORR
		A	B	C, D, E			
Diabetic dataset	**MLP**	**78.889**	**21.111**	**0**	**3.784**	**2.976**	**0.745**
	SVR	77.419	22.581	0	4.014	3.162	0.711
	RFR	78.495	21.505	0	4.010	3.181	0.712
	XG Boost	77.419	22.581	0	3.862	3.140	0.737
Non-diabetic dataset	**MLP**	**93.239**	**6.761**	**0**	**0.953**	**0.753**	**0.472**
	SVR	91.573	8.427	0	0.956	0.750	0.466
	RFR	91.854	8.146	0	0.972	0.771	0.443
	XG Boost	90.309	9.691	0	1.012	0.800	0.390
Continuous dataset	MLP	91.176	8.824	0	0.983	0.753	0.469
	SVR	90.809	9.191	0	0.995	0.769	0.448
	RFR	91.176	8.824	0	1.011	0.783	0.432
	XG Boost	90.809	9.191	0	1.037	0.799	0.413
	**STMF-LSTM**	**95.089**	**4.911**	**0**	**0.811**	**0.620**	**0.678**

**Table 5:** Results of different ablation experiments.

Method	Inputs	Model	Parkes EGA (%)			RMSE (mmol/L)	MAE (mmol/L)	CORR
			A	B	C, D, E			
A	PPG	MLP	90.441	9.559	0	1.016	0.792	0.429
B	PPG + Δ*A*	MLP	91.176	8.824	0	0.983	0.753	0.469
**C**	**PPG + Δ*A* + Glu**	**STMF-LSTM**	**95.089**	**4.911**	**0**	**0.811**	**0.620**	**0.678**

**Table 6:** Comparison of proposed method with other studies.

Method	Principle	Model	RMSE (mmol/L)	MARD (%)	Glucose Range (mmol)
Kim et al., 2020 [21]	Glucose	GRU	1.19	11.1	3.3–22.2
Ali et al., 2024 [33]	PPG	CBHFF	1.67	17.88	-
Du et al., 2025 [18]	PPG	CBGnet	0.476	**5.13**	4.4–6.6
Chowdhury et al., 2024 [34]	PPG + EDA + ST + food features	MMG-net	0.959	12.57	-
Ours	PPG + Δ*A* + Glucose	STMF-LSTM	0.811	**10.122**	3.9–9.3

## Data Availability

Data are unavailable due to privacy or ethical restrictions.

## Appendix A

Repeated experiments were performed on three subjects, with each subject conducting 10 consecutive measurements under the same conditions. The type A uncertainty of the PD sensors was estimated by calculating the standard deviation (SD) of the measured differential absorbance. As shown in Table A1, the maximum type A uncertainty of the six differential absorbance values was 0.026.

**Table A1:** Type A uncertainty of the PD sensors.

Differential Absorbance	Standard Deviation			Standard Deviation of Combined Samples
	Subject A	Subject B	Subject C	
Δ*A_near−far_* (625 nm)	0.005	0.006	0.009	0.007
Δ*A_near−trans_* (625 nm)	0.005	0.004	0.010	0.007
Δ*A_near−far_* (850 nm)	0.023	0.017	0.032	0.024
Δ*A_near−trans_* (850 nm)	0.021	0.019	0.035	**0.026**
Δ*A_near−far_* (940 nm)	0.012	0.020	0.014	0.016
Δ*A_near−trans_* (940 nm)	0.011	0.022	0.015	0.016

## Appendix B

**Table A2:** Specifications of the blood glucose reference instrumentations.

Parameters	Abbot’s Freestyle Libre	OneTouch Verio Flex
Measuring range	2.2~27.8 mmol/L	1.1–33.3 mmol/L
Resolution	0.1 mmol/L	0.1 mmol/L
System accuracy	93.2% ^1^ within ±1.11 mmol/L or ±20%	99.5% ^2^ within ±0.83 mmol/L or ±15%

^1^ A total of 146 subjects with diabetes wore two CGM sensors for 14 days. Venous blood glucose values were measured by Yellow Springs Instrument Life Sciences 2300 STAT Plus™. A share of 93.2% (17635/18926) of the results were within a deviation of no more than ±1.11 mmol/L when the test range was <4.4 mmol/L, and no more than ±20% when the test range was ≥4.4 mmol/L [35]. ^2^ Blood samples collected from 100 patients were tested using the OneTouch Verio Flex blood glucose monitoring system and the YSI 2300 Glucose Analyzer. A share of 99.5% (597/600) of the results were within the allowable deviation, which was no more than ±0.83 mmol/L when the test range was <5.55 mmol/L, and no more than ±15% when the test range was ≥5.55 mmol/L [36].
